# Home-Based Interventions to Treat and Prevent Childhood Obesity: A Systematic Review and Meta-Analysis

**DOI:** 10.3390/bs9040038

**Published:** 2019-04-12

**Authors:** Rian Adi Pamungkas, Kanittha Chamroonsawasdi

**Affiliations:** 1 Department of Nursing, Faculty of Health Sciences, Esa Unggul University, Jakarta 11510, Indonesia; rian.adi@esaunggul.ac.id; 2 Department of Family Health, Faculty of Public Health, Mahidol University, Bangkok 73170, Thailand

**Keywords:** childhood obesity, home-based intervention, meta-analysis

## Abstract

Childhood obesity has adverse impacts on premature mortality and morbidity. Managing obesity could prevent premature mortality and several types of complications among high-risk groups. This study aimed to review and examine the effects of home-based interventions to treat and prevent childhood obesity. Three databases, i.e., PubMed, Scopus, and Science Direct, were included to extract articles related to the topic. The terms “childhood obesity”, “home-based intervention”, “parental program”, and “parental involvement” were used as the primary keywords. Appraisal of the systematic review was based on PRISMA formats. Of 1556 publications identified, 22 studies fulfilled the inclusion criteria and were appropriate to conduct a meta-analysis. Overall, the home-based interventions reduced the body mass index (BMI) z-score by 36.99% (z = 36.99, *p* = 0.00). The data analysis indicated considerable heterogeneity among all interventions (Chi-square = 926.41, df = 22 (*p* < 0.000001), I^2^ = 98%). The home-based intervention positively reduced BMI. Our findings could guide future meaningful home-based interventions to treat and prevent childhood obesity.

## 1. Introduction 

Childhood obesity has become a crucial problem in both developed and developing countries. In 2018, the World Health Organization [1] estimated 41 million infants and children were obese. This number is predicted to increase to 70 million by 2025 [1].

Childhood obesity has adverse effects on premature death as well as on physical and psychosocial problems of the child [2]. The increasing epidemic is associated with unhealthy eating habits, low physical activity, and sedentary lifestyle during childhood [3].

Children are well-recognized as a priority target group of obesity interventions globally. Without proper intervention to prevent and manage childhood obesity, obese children will face life-long chronic illnesses including cardiovascular disease, diabetes, and cancer [4]. Dealing with childhood obesity is the cornerstone for preventing long-term complications and reducing premature death from chronic non-communicable diseases (NCDs). The American Academy of Pediatrics has recommended managing nutritious diets and physical activity. Moreover, limiting sedentary time such as that used for watching television, playing video games, using a computer for entertainment, and other screen-based activities should be emphasized [5]. From the literature review of Mulrine concerning interventions to prevent childhood obesity using different systematic reviews, the findings indicated a combination of strategies involving multiple settings such as the school and family are the most effective ways to combat the problem [6]. Measures of primary prevention by facilitating healthy environments to enhance healthy lifestyles of normal weight children together with secondary prevention to decrease body weight within its normal range among overweight and obese children were used in both school-based and community-based programs, with parental involvement as a focal point to monitor and facilitate children’s healthy behaviors.

Parents constitute key persons who influence their child’s behaviors [7]. Parents are powerful agents of change in managing childhood obesity because they can manage the child’s weight-related behaviors by promoting regular feeding to their child and discussing healthy behaviors, as well as establishing a healthy home environment [8]. In summary, the parental role is considered a fundamental factor to prevent and control childhood obesity.

The home-based approach may improve parental skills and self-confidence with respect to managing healthy eating habits, increasing physical activity, preventing sedentary lifestyles, and promoting healthy lifestyles among their children [8]. Engaging parents in facilitating treatment and prevention of childhood obesity will lead to reduced risk of NCDs and its complications among the childhood period [2].

Family is the closest environment to the child. Parents play a vital role in childcare and child rearing. The home atmosphere is important to facilitate childhood development. Most children spend most of their time conducting activities at home such as eating, playing, and watching television. Parental roles in preparing meals at home and parental feeding styles are major contributing factors to child eating behaviors [5]. Because family is important, a home-based intervention is crucial. The home-based intervention program has been proved as an effective strategy to manage child’s health behaviors related to obesity [6].

Several studies on childhood obesity treatment and prevention based on a home-based approach showed positive effects on body mass index (BMI)z-score reduction (BMI= Body weight in kilogram divided by Height in square meter) and weight-related behavioral change among children [8,9]. Among the parents, more parental and maternal outcomes in behavioral changes of avoiding sugary beverages were found compared with before the implementation period [10]. In addition, a home-based intervention program was also significant in reducing blood pressure and improving the quality of life and eating behaviors [11], and reducing television watching and consumption of snacks and sweets [12]. The parental role can be used as guidelines to prevent and control childhood obesity. Though the benefit of the parental-based program is commonly discussed in the original article, few studies have explored impacts of the home-based approach by both systematic review and meta-analysis with respect to childhood BMI z-scores. A meta-analysis of home-based interventions needs to be explored to prove fundamental strategies on treating and preventing childhood obesity by improving child health behaviors and parental feeding behaviors. Fruitful findings from this study could support and strengthen home-based interventions in treating and preventing childhood obesity in different contexts.

## 2. Objective

The study is aimed at reviewing and examining the impacts of home-based interventions on treating and preventing childhood obesity.

## 3. Methods

### 3.1. Data Sources

Three databases, including PubMed, Science Direct, and Scopus were used to extract relevant articles. These articles were selected based on the inclusion criteria and followed the Preferred Reporting Items for Systematic Reviews and Meta-Analysis (PRISMA) framework for critical appraisal of each article. Home-based intervention was used as a primary search term in each article title and finally 22 articles fit the meta-analysis (See Appendix A).

### 3.2. Search Strategy 

Several keywords were used to obtain relevant articles used in this review, comprising “home-based intervention”, “parental program”, “childhood obesity”, and “young age obesity”. Available articles related to the home-based interventions as well as parental programs for childhood obesity prevention were reviewed to extract information. To obtain the current articles, relevant to childhood obesity treatment and prevention programs, the period of publication was limited to ten years between 2009 and 2018. We used the Participant–Intervention–Comparison–Outcomes (PICO) format to design the criteria of articles for meta-analysis as follows; 

P: Childhood, school age 

I: Home-based intervention, parental-based intervention 

C: Control group 

O: Physical activity, sedentary lifestyle and healthy diets

  BMI z-scores, changes in obesity and overweight status summarized as proxy indicators

### 3.3. Inclusion Criteria of Study

The inclusion criteria of this study comprised: (1) English language articles published between 2009 and 2018; (2) randomized control trial (RCT) design with nonequivalent control group; (3) home-based or parental-based intervention to improve health behaviors and health outcomes; (4) BMI z-score measured as the proxy outcome; and (5) reported data for one or more specified outcomes such as BMI change before and after implementation or BMI z-score.

### 3.4. Exclusion Criteria

The researchers excluded studies based on the following inclusion criteria: (1) not being a home-based or family-based program; (2) type of study including descriptive, qualitative research, one-group quasi-experimental design, mixed-methods without testing the program effect, and quasi-experimental study with two groups pretest and posttest design with nonequivalent control group; (3) intervention target focused on adult population; (4) not focused on obesity treatment and prevention; and (5) published in a dissertation format or review studies such as literature review, concept analysis, systematic review, and meta-analysis, not involving home-based interventions for treating and preventing childhood obesity.

**Figure 1:**
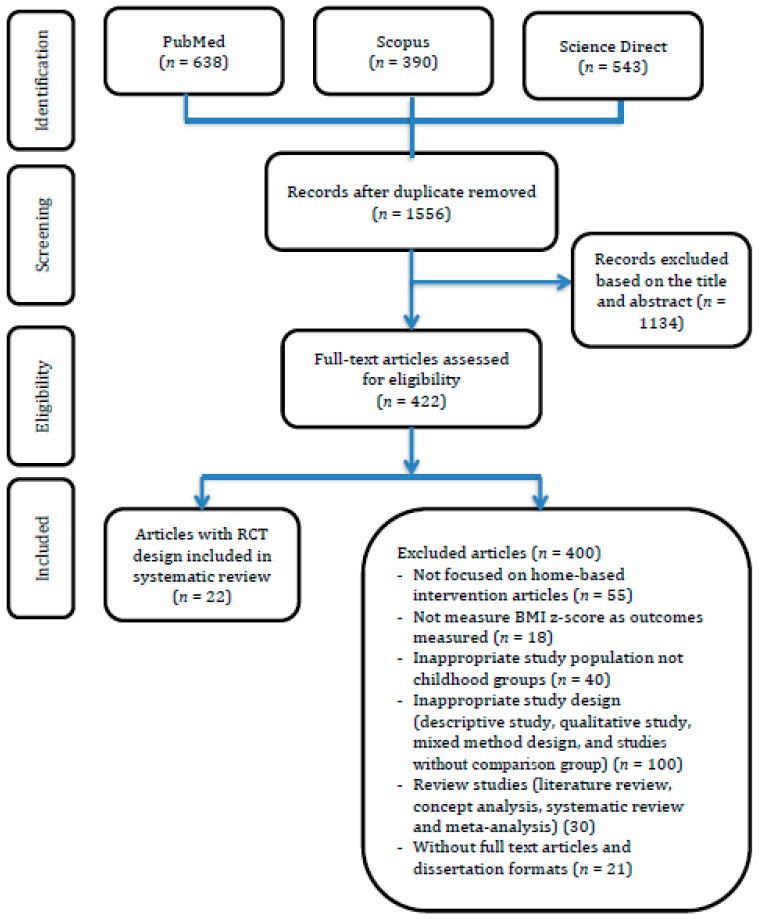
Summary of evidence-based searching process and selection criteria. RCT: randomized controlled trial; Body mass index (BMI).

Details of search strategies, eligibility articles and included articles selected to review and to analyze in this study are summarized as Figure 1.

### 3.5. Quality Assessment and Controlling the Risk of Bias

The Consolidated Standards of Reporting Trials (CONSORT) [13], a validated scale for intervention studies in meta-analysis, was used to perform quality assessment and control risk of bias found in each study. The quality assessment items comprised: (1) random adequate sequence generation (selection bias); (2) allocation adequately concealed (selection bias); (3) blinding of participants and personnel (performance bias); (4) blinding of outcome assessment (detection bias); (5) incomplete outcomes data (attrition bias); and (6) selective reporting (reporting bias) and other biases.

The risk of bias assessment tool from the Cochrane tool was used as the criteria for judging the risk of bias. We assigned the risk of bias scales consisting of low, high, and unclear risk. When the study had several adjustment models, we extracted all information that reflected the maximum adjustment level to control the risk of bias.

### 3.6. Statistical Analysis

Meta-analysis was conducted using Comprehensive Meta-analysis (CMA), Version 3.0 to analyze by pooling data from each study. The effectiveness of the home-based intervention program on BMI change between the experimental and control groups was explained using mean different and standard deviation. All data should be considered as continuous and thereby to determine outcomes measured, a mean difference with 95% confidence interval was also presented in the analysis process. Heterogeneity of variance was explained using chi-squared and I^2^ index tests. The random-effects model was performed when the chi-squared for the heterogeneity test was insignificant. Statistical analysis with standard *p* < 0.05 was carried out by the review manager and a forest plot was carried out to describe the potential publication bias.

## 4. Results

### 4.1. Study Literature 

We extracted three databases to screen and select articles based on the PRISMA flowchart and found 1556 documents had been published from 2009 to 2018. The researchers verified relevant articles related to home-based interventions to treat and prevent childhood obesity based on the titles and abstracts of the selected articles. Thereby, 1134 articles were excluded due to inconsistent inclusion criteria.

After screening articles based on research titles and abstracts, we recruited 422 eligible articles with full texts of publication. Based on the inclusion criteria, only 22 articles fulfilled the requirements to be examined in the meta-analysis phase. On the other hand, more than 400 articles were excluded for several reasons as described below. In all, 55 studies did not design the intervention using a home-based approach, instead using a school-, community- or health center-based method without family or parents as a focal point. A total of 69 studies measured outcomes using different indicators rather than BMI changes or BMI z-score, which were not included as primary outcomes in the meta-analysis. Exactly 40 population-based studies focused on other chronic diseases which were unrelated to childhood obesity such as hypertension, diabetes mellitus, and other metabolic disorders. In addition, obesity with comorbidities was also excluded in these reviews. Because these reviews focused on an RCT study design with pretest and posttest, 100 studies were excluded due to inappropriate study design such as descriptive study, qualitative study, mixed method design, and studies without comparison groups, while 30 studies were presented in a review format including literature review, concept analysis, systematic review, and meta-analysis. Other reasons to exclude 21 articles in this meta-analysis were no full texts available and publication in a dissertation format.

### 4.2. Controlling Risk of Bias

Of 22 studies included in this review, most were verified as having low risk of bias because most of these studies employed the blinding technique to avoid selection bias during sample selection and treatment allocation. The blinding technique was also used to avoid measurement bias after implementing the home-based intervention program.

### 4.3. Home-Based Intervention Features

#### 4.3.1. Parental Involvement

As parents are key individuals in the family, incorporating parents as a part of the childhood obesity intervention is necessary. Parental participation in the management of childhood obesity leads to more favorable results to treat and prevent obesity when families positively influence healthy eating styles and physical activity of their children. Parents are usually responsible for preparing food, for suggesting proper food to eat when the child is away from home, and for creating a social environment fitting the child’s needs. They also influence the physical activity of the child.

This review focused on home-based intervention to treat and to prevent childhood obesity among children and early adolescents. In general, programs were offered and designed for individual counseling on how to manage and control healthy food, enhance physical activity, and avoid a sedentary lifestyle. Moreover, key strategies also included goal-setting, problem-solving, and parent–child communication. Parents have roles to support their child’s healthy behaviors.

Altogether, 23 studies indicated effective parental involvement to improve health outcomes among their children. All home-based interventions provided home learning by encouraging parents’ active participation in the learning process followed by individual and group discussions [14,15]. To improve parental skills on childcare and child rearing, some studies offered intensive programs on modeling therapies and engaged parents in long-term plans using problem-solving skills to combat the child’s barriers [9]. Some studies focused on efficient time parents spent on their child for emotional support with admiration when the child was doing well, on parents’ efforts to limit child’s sedentary behaviors, and that spent on managing their child’s improper behaviors [16].

Some studies emphasized positive parental skills such as managing healthy behaviors by keeping track of their child’s behaviors [8], and establishing goal setting and effective plans to promote a healthy home environment [17]. Another study incorporated social support and encouraged availability and accessibility of healthy foods in the home environment to promote healthy eating behaviors [18].

#### 4.3.2. Nutrition Strategies

One study indicated the implications of an energy balance model to prevent and control childhood obesity by reducing fat and sugar intake while increasing fruit and vegetable intake to decrease the risk of weight gain [19]. Most obesity prevention programs recommended consuming low-fat and high-fiber diets; however, this review not only described kinds of food intake but also manipulated dietary change as a part of the intervention, such as managing the portion size or snacks to eat daily. Another strategy was to manipulate the food environment at home because the food environment plays a vital role in treating and preventing childhood obesity.

In general, home-based approaches related to the nutrition strategies were incorporated at home. The home environment influenced the child’s eating behaviors the most. Moreover, parental styles also helped develop childhood healthy lifestyles [20,21].

Various strategies related to nutrition approaches were conducted in this review. Adequate information on healthy and balanced diets is essential for both children and their parents. Some studies explained positive ways to manage a balanced diet by increasing knowledge on healthy food intake, avoiding eating snacks, reading instructions, and understanding labels on food and drink, as well as modifying recipes [8,9,17,22,23]. Arauz’s study introduced interactive nutrition games such as Jeopardy, indoor jump rope, and food pyramid bingo to improve nutritional balance and to prevent boredom [15].

To improve healthy eating behaviors among children and their parents, ultimate knowledge is less important than managing the enormous pressures needed to achieve proper weight. This included reducing snack intake, encouraging consuming a proper diet [11], increasing vegetable and fruit consumption, modifying meal and snack preparation [17,24,25], and encouraging children to consume less high energy foods such as fatty meat, fried snacks, and sweetened drinks [26].

In the study of van Nassau et al., the Dutch Obesity Intervention in Teenagers (DOiT) Program focused on raising awareness and changing behaviors to prevent childhood obesity among children and parents by reducing sugar-containing beverage (SCB) intake. This study aimed at lowering high-energy intake, reducing snack and sweetened food intake, and encouraging high fiber and low fat intake at daily breakfast. Social support was provided to influence self-confidence of parents in managing childhood obesity and strengthening availability and accessibility of health products at home environment [18].

Another study was conducted using mailed interactive kits and telephone coaching to improve parental skills and child’s behaviors including decreasing sugary drinks and fast food, increasing fruits and vegetables, and preparing meals at home. These skills also comprised setting routine sleep and meal times, creating a supportive home environment, promoting parental role models of healthy eating as well as improving parental feeding styles to maintain their child’s weight [10].

Arranging appropriate nutrition education concerning food standards to enhance parental skills on preparing food within a home environment would help parents to provide healthy food for their children. Moreover, interventions also encouraged parents concerning behavioral changes related to obesity prevention and motivated their self-efficacy in dealing with their child’s problems and in managing their child’s healthy diets.

#### 4.3.3. Increased Physical Activity 

Increased frequency of physical activity was considered an implication of the energy balance model for people with obesity. Traditional approaches focused only on raising awareness and behavioral change to reduce physical inactivity. Recently, social, physical, and cultural environments have become crucial determinants of the extent to which one is active [27]. Family support has a positive effect on the child’s physical activity. A family-based obesity prevention program by enhancing physical activity is the most effective strategy for combating childhood obesity [28].

The Canadian Pediatric Society (CPS) encourages performing physical activities and sports at least 30 min daily, with at least 10 min involving vigorous activities for children and adolescents to prevent and control childhood obesity. To conduct effective programs to promote physical activity at home, parents must be encouraged to serve as role models to their children on performing safe physical activities that all family members can enjoy [27,29].

Home-based approaches for physical activity have been conducted in some studies. The importance of physical activity is known as an essential component in Diabetes Mellitus Self-management (DMSM) Programs. The implementation of frequent and fun exercises among young people should emphasize performing regular and appropriate physical activity and appropriate physical activity at home.

In this review, some studies identified the effects of physical activities on reducing childhood obesity. The activities comprised aerobics [22,30], sports exercise [8,18], and sensory-motor and physical activity, with warming-up before and cooling down after exercise [25]. Another study was conducted to promote enjoyable physical activities such as dancing, team building, and playing ball games to avoid boring activities [31].

Farias et al. designed a home-based intervention to increase physical activity. The aims of this study were focused on raising awareness and promoting behavioral changes by educating physical exercise classes to improve knowledge and skills of children concerning physical activity [32]. Physical activity with heart rate monitoring was conducted after setting goals, strengthening positive support and providing positive reinforcement to the child. Among the parents, enhancing problem-solving skills to increase their self-confidence in treating and preventing childhood obesity and encouraging the availability and accessibility of playground for physical activity at home were included [11,24].

Several shreds of evidence showed regular physical activity have a positive effect on reducing weight and improving insulin sensitivity [31]. Each kind of aerobic exercise significantly reduced systolic and diastolic blood pressure among children 9 to 11 years old [32]. Resistance training such as weight lifting could prevent hypertension among adolescents [33]. Regular physical activity also benefits all youth regardless of weight. It was associated with an increase in their self-esteem and self-concept and a decrease in anxiety and depression [34].

#### 4.3.4. Reduction of Sedentary Behaviors

The sedentary lifestyle has been proven to exhibit a causal relationship with obesity and overweight status. Research has been conducted all over the world to find out modifiable risk factors for current life style weight control. Reducing sedentary behavior implies an energy balance model to treat and prevent obesity. Encouraging the child to be involved in other physical activities such as housework and sports can reduce sedentary behaviors such as watching television and playing video games. The theory of the Information–Motivation–Behavioral (IMB) Model has proved that parents should provide sufficient information to their child to support regular practices among their children concerning healthy diet and sufficient physical activity to control children’s weight [33].

In this review, some studies focused on reducing sedentary behavior to manage childhood obesity [10,11,12,15,18,24]. The evidence showed a positive effect on various markers that included reducing both morbidity and mortality [34]. The American Academy of Pediatrics (2001) [35] has recommended guidelines to reduce sedentary lifestyle by limiting time of watching television, removing media gadgets from children’s bedrooms, and monitoring and viewing program contents along with the children. Reducing time of watching television is considered as one of the most modifiable causes of obesity in children.

### 4.4. Effectiveness of Home-Based Interventions on Health Outcomes 

We recruited 22 studies in this review based on the inclusion criteria to determine the effectiveness of the intervention. Summary findings of the home-based interventions on health outcomes between the intervention and the control groups are presented below. 

#### 4.4.1. Body Weight

This review examined the effect of home-based interventions on reducing child weight. Regard 22 existing studies; about 73% produced a positive impact of the home-based intervention with respect to changed BMI z-score as a primary outcome of the intervention [8,9,11,14,17,18,22,23,25,26,30,31,32,36,37,38,39]. Six studies confirmed no significant changes in BMI z-scores between the intervention and the control groups after receiving the home-based intervention [10,12,15,24,39,40].

#### 4.4.2. Healthy Eating Behaviors

Lifestyle behaviors are fundamental approaches to control weight among obese children. Twenty-two articles examined the impacts of home-based interventions concerning self-care behaviors. Substantial evidence proved that healthy lifestyle behaviors, including healthy diet, active physical activity, and reducing sedentary behaviors had impacts on reducing weight among overweight and obese children.

Four studies have reported a positive impact on reducing high-fat food and sugary beverages after receiving the home-based intervention [24,36,40,41]. One study showed appropriateness in selecting healthy diet and increasing fruits and vegetables intake [9,10,24,38,41], while two studies improved attitudes concerning eating patterns by reducing snacks and maintaining a balanced diet [11,37] as well as increasing daily fruit servings [26] and decreasing soft drink consumption [9]. All of those studies showed a high level of parental roles in managing healthy diets for overweight and obese children.

#### 4.4.3. Physical Activity and Sedentary Lifestyle

Seven studies also confirmed a significant increase in level of physical activity after participating in home-based intervention programs [9,10,17,24,25,37,39,40]. Four studies focused on modifying behaviors to reduce sedentary lifestyle including reducing the time of watching television or playing computer games [10,12,24,25]. On the contrary, one study showed no significant change after receiving the program [15].

### 4.5. Summary of Effects Analysis of Interventions

**Figure 2:**
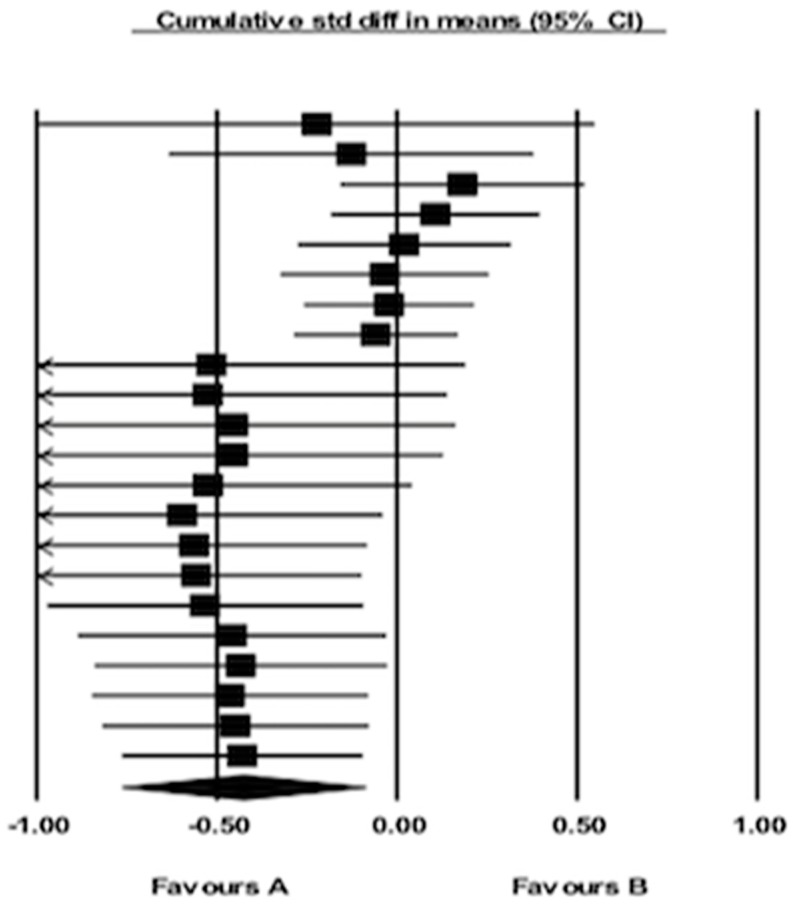
Forest plot for BMI z-scores.

**Figure 3:**
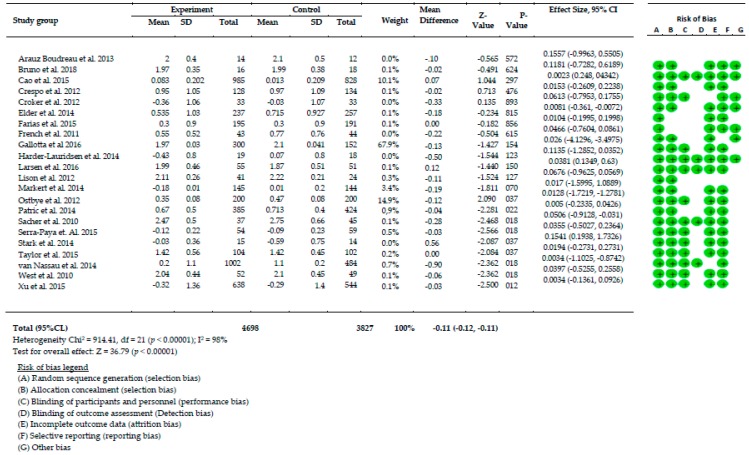
Meta-analysis results.

Twenty-two randomized control trials were involved in this meta-analysis to examine the effects of home-based approaches concerning change of BMI z-scores. Figure 2 demonstrated on cumulative effects in BMI-Z scores of changed among 22 studies using Forest plot. Data analysis from Figure 3 shows considerable heterogeneity among all interventions (Chi-square = 926.41, df = 22 (*p* < 0.000001), I^2^ = 98%). The effect sizes of 22 interventions were ranged between 0.0034-0.1557. Therefore, the overall effect of home-based approaches significantly reduced BMI z-scores by approximately 36.99% (z = 36.99, *p* < 0.001).

## 5. Discussion 

We conducted a systematic review and meta-analysis of 22 existing studies related to home-based interventions for childhood obesity prevention and control between 2009 and 2018. The findings of this review confirmed the effectiveness of home-based interventions on reducing weight. A randomized control trial was employed as a robust design in every study.

The home-based intervention was generated in the home setting to support children and their parents for managing obesity. This strategy offered a combination of didactic teaching and interactive or participatory learning approaches followed by goal setting, action planning, problem-solving, positive reinforcement, and monitoring of child behaviors.

This review found that home-based approach used collaborative strategies across studies including promoting healthy diets, increasing physical activity and reducing sedentary behaviors. Parental roles were incorporated in the program activities including managing food intake and recognizing the portion size, as well as enhancing problem-solving skills on managing barriers while implementing the program. Increasing the frequency of physical activity and reducing sedentary hours were achieved using fun strategies to minimize boredom. Many studies also described the details of parental involvement in the program activities such as providing emotional support, problem-solving and helping their children to manage their diets, facilitating physical activity, managing their child’s emotional distress, and providing information for weight control. In general, parental roles in the home-based interventions focused on how to facilitate, accommodate, remind, motivate, and coach their child’s appropriate behavior changes.

Findings from this study showed a significant reduction in body mass index (36.79%) after receiving the home-based intervention. This study was quite similar to a related meta-analysis conducted using a family-based program for childhood obesity prevention, which found a significant reduction on weight and improvement of other behavioral outcomes such as eating behaviors, physical activity, and sedentary lifestyle after receiving the program [42]. Another meta-analysis showed that family-based interventions targeting childhood obesity were successful in reducing weights both short and long term. This study also reported a moderate to large effect size for change in the target outcomes on child’s BMI (BMI percentile, BMI z-scores, percent overweight) after participating in the family-based intervention [43].

Moreover study conducted by Bleich et al. designed the combination of school based intervention with home element had a significant effect on preventing obesity [44]. Another meta-analysis also showed that the home-based intervention had a positive effect on weight outcomes [45]. However, the strength of this evidence is low to support the effectiveness of home-based child obesity prevention programs. Therefore, the recent meta-analysis focused on home-based intervention, particularly those incorporating the parenting strategies for treatment and prevention of childhood obesity.

## 6. Strengths and Limitations

This meta-analysis focused on randomized control trials (RCTs) to prevent and control childhood obesity. The aim was to test the effectiveness of the home-based interventions concerning reducing child’s weight. This design was considered as rigorous method to ensure the validity of health outcome measures. Another strength was the focus on data pooling of the various studies by analyzing BMI z-scores to examine the effect of outcome measures. The random effects model was used in the analysis to verify the distribution of true effect sizes by estimating the mean distribution of pooled data among all 22 studies. To ensure the up-to-date and relevant data of the articles, this review focused on articles published between 2009 and 2018. Also, these studies analyzed the risk of bias with almost 98% heterogeneity for each study. Although we created this systematic review by hand tracking, there may have been some studies related to home-based interventions for childhood obesity prevention that remained unidentified.

## 7. Conclusions

Developing a home-based intervention to prevent and control childhood obesity is an integral part of weight reduction, focusing on improving child health as well as parental behaviors. In conclusion, this systematic review and meta-analysis found that home-based interventions could reduce BMI z-scores among obese children.

## Appendix A

**Table A1:** Home-based intervention for childhood obesity.

Author, Year	Target Group Intervention	Focused Activities			Home-based Program	Instrument	Outcomes
		(DB)	(PA)	(SB)			
Araus Boudreau, 2013 [15]	Both the child and parents	*	*	*	**Power up classes including** - Educated children and caregivers about health behaviors including nutrition, physical activity, and stress management - Encouraged practice through interactive games and activities such as Jeopardy, in-door jump-rope, and food pyramid bingo - Group discussion about portion control, healthy snacking, label reading, goal setting and watching TV - Reinforcement to promote PA at home **Health coaching** - Coaching to empower families to incorporate learning behaviors and to address both family and social barriers - Home visit or phone meeting at least every month	- BMI z-score using SAS for CDC growth charts - SPAN questionnaire	- The intervention group showed non significant difference for metabolic markers of obesity, BMI z-score, and PA
Bruno, 2018 [22]	Both the child and parents	*	*		- Provided education for children and their parents about the benefits of the Mediterranean diet and exercise - Physical exercise program including a training circuit with increasing intensity, compromising anaerobic activity - Print-based group used a written guide	- BMI z-score	- The intervention group showed a significant effect for reducing the BMI score
Cao, 2015 [23]	Both the child and parents	*	*		- Health education program on obesity prevention - Parent–school meeting on obesity prevention every semester - Provided information about balanced diet, its principles, and methods - Instructions to parents on healthy eating habits of children - Jump rope provided to each student and appropriate level of physical activity at home - Extracurricular activities during vacations	- BMI z-score	- The intervention group had significantly lower odds of developing obesity and had decreased average BMI z-score
Crespo, 2012 [40]	Both the child and parents	*	*	*	- Home visits to discuss barriers on healthy eating and PA, ways to prepare a meal plan at home, benefits of promoting healthy diet and PA for children - Booster phone calls for goal setting and monitoring the progress - School playgrounds and salad bars - Physical education equipment and children’s menu at restaurants - Posters, newsletters, frequent produce buyer cards in grocery stores	- BMI z-score - 26-item parenting scale - 30-item behavioral strategies	- The intervention group showed non significant difference on BMI - The intervention group showed a significant difference in healthy eating and PA as well as parents support
Croker, 2012 [11]	Both the child and parents	*	*		- Self-monitoring on food and activity diaries - Goal setting, positive reinforcement, stimulus control and relapse prevention to modify behaviors - Asking parents to support their child’s behavioral changes and make the home environment to encourage family-wide uptake of healthy lifestyle behaviors - Reduced snacks and encouraged consumption of balanced diets - Reduced time spent on sedentary behaviors	- BMI z-score - Children’s eating attitude test	- The intervention group had significantly lower odds of developing obesity and had decreased average BMI z- score. - The intervention group showed a significant improvement in eating attitude
Elder, 2014 [24]	Both the child and parents	*	*	*	- Telephone survey about families’ recreation centers - Telephone consultation to promote a healthy lifestyle, and problem-solving of challenges - Individualized to self-monitoring and goal setting. - Nutrition behaviors targeted by increasing vegetables and food intake, and modified in meal and snack preparation, increasing healthy portions by modified food consumption, reducing screen time and avoiding eating while watching TV, and increasing meals eaten together with family members - Enhanced physical activity by increasing the amount of moderate to vigorous PA, availability and accessibility of PA, and variety of fun and frequent PA	- BMI z-score - Actigraph accelerometer - 21-item PACE	- The intervention group showed non-significant different on BMI and child waist circumference compared with the control group - The intervention group showed consumption of less high-fat food and fewer sugary beverages - The intervention showed a significant difference in PA and sedentary time
Farias, 2015 [32]	Child-only		*		- Physical education classes weekly - Participants in the control group performed the usual physical activity - Participants in the experimental group completed the physical activity with heart rate monitoring comprising aerobic activity [exercise for flexibility, muscular strength, jumping rope, walking, alternating running, continuous jumping, recreational games, sports games (volleyball, soccer, handball), and stretching	- BMI z-scores	- The program showed a positive effect on body composition and physical activity in the experimental group
French, 2011 [12]	Parents only	*	*	*	- Education sessions for parents on eating, PA, and sedentary reduction for children - Limited fast food and increased fiber intake. - Guidelines for parents on how to control the child’s weight - Promoted 30 min/day exercise	- BMI z-score - The modified food frequency questionnaire - International PA questionnaire - Self-reported TV viewing	- The intervention group showed a non-significant effect on BMI score after receiving the program - The intervention reduced watching TV and eating snacks/sweet sweetened food
Gallota, 2016 [25]	Both the child and parents	*	*	*	**Healthy Nutrition Intervention** - Increased consumption of fruits and vegetables - Conducted short lectures/talks, games, and sensory workshop - Supported establishing healthy eating habits - An information campaign for parents and distributing informative materials **Physical Education Intervention** - Involved children to perform the PA with 15 min of warm up, 35 min of moderate to vigorous PA, and 10 min of cool down - Designed a program to promote health, fitness, sensory-motor skills, social and communicative development - Developed psychomotor competence and expertise in movement-based problem solving	- BMI z-scores - Parental proxy interview for sedentary time - Physical activity questionnaire	- The intervention group showed significant difference on PA, sedentary behaviors, and specific food consumption after receiving programs
Hander-Lauridsen, 2014 [36]	Both the child and parents	*			- A weekly group training session - A 90-minute weekly group training session with the children, their parents, and siblings - Individual nutritional guidance and coaching of the children and their families - Cooked and dined with the children and their families	- BMI z-score - Dutch Obesity Intervention in Teenagers (DOiT) questionnaire for measuring behaviors	- The intervention group showed a significant BMI score - The intervention group reduced consumption of sugar-containing beverages consumption
Larsen, 2016 [31]	Both the child and parents	*	*		- Engaged exercise with a focus on PA enjoyment and motivation such as dancing, team building, and alternative ball-games - Health classes focused on behavioral changes and information regarding healthy cooking in the household and advice on how best to support the child’s health behaviors - The family-based intervention that is focused on families discussing and sharing experiences related to a chosen topic - Meeting for encourage and motivate the children on behavioral changes	- BMI z-score	- The intervention group showed a significantly lower BMI z score after 52 weeks of interventions compared with the control groups.
Lison, 2012 [30]	Both the child and parents		*		- Moderate aerobic activity with warming up and cooling down (stretching) - Tailored resistance training such as abdominal curl-ups, prone hip extensions, and wall push-ups squats and bicep curls. - Positive environment with a positive feeling and attitude towards PA - HOX group participants which involved both resistance and aerobic training exercise - Received the detail of written instructions - Provided a daily exercise log book for six months of exercise	- BMI z-scores	- The intervention group showed a significantly lower on BMI score after 24 months follow-up
Markert, 2014 [37]	Both the child and parents	*	*	*	- Telephone counseling programs. - Interview contents about obesity and associated co-morbidities, dietary habits, eating behavior, physical activity and leisure time behaviors, physiological support, and stress management - Two free coaching telephone sessions and evaluation of the program.	- BMI z-score - Food frequency questionnaire and family eating habit questionnaire - PA scale	- The intervention group showed a significant difference in BMI z-score - The intervention showed a positive effect on eating patterns, food consumption, and PA
Østbye, 2012 [10]	Both the child and parents	*	*	*	- Participants received a mailed interactive kit and telephone coaching session - Parenting skill instructions emphasized by an authoritative parenting style, routines for sleep and mealtimes, supportive home environment, role modeling of healthy eating and PA, and improved feeding style - Education about healthy behavioral changes included decreasing sugary drinks and fast foods, increasing fruit and vegetable consumption, meals prepared at home, moderate to vigorous PA, and reduced sedentary behavior - Addressed motivation, self-efficacy, and barriers to change	- BMI z-score - 24-hour dietary recall - Article accelerometers	- The intervention group showed a non-significant difference on BMI, reducing instrumental feeding, eating the snacks, and watching TV, and increasing fruit/vegetables intake after receiving the program - The intervention group showed non-significant difference in PA and sedentary time
Patric, 2013 [38]	Both the child and parents	*	*		The intervention group received interventions including - A computer-generated assessment of diet and physical activity behaviors that included an action plan with goals corresponding to their stage of readiness (Goal Sheet) - Two brief counseling sessions with their primary physician and a stage-matched educational manual (Teen Guide) - Monthly calls from a health counselor, - Monthly mailed tip sheets and guidance and support from their parents, which was family-based	- BMI z-score - 7-day PA recall - Validated survey for measuring sedentary time	- The intervention group showed a significant difference in BMI, improved consumption of fruits and vegetables
Sacher, 2010 [17]	Both the child and parents	*	*		- Nutrition education comprised of healthy eating advice for obese children, instructions on the reading and understanding food and drink labels - Families took part in a guided supermarket tour and were given healthy recipes to try at home - Preparation on healthy meals and fruit and vegetables - Behavioral change session included stimulus control, goal setting, reinforcement, and response prevention to establish a healthy-promoting home environment - Exercise session focused on noncompetitive group play and the importance of managing and reducing weight	- BMI z-score - Non-validated questionnaire for measuring the level of PA and sedentary behaviors	- The intervention group showed a significant difference in BMI after receiving Mind, Exercise, Nutrition, Do it Program - The intervention group showed positive effects on physical activity, reduced on sedentary behaviors, and increased self-esteem
Serra-Paya, 2015 [39]	Both the child and parents	*	*	*	- Supervised physical activity session for children about 90 one-hour sessions - A family theoretical and practical session for parents to give the family the opportunity to exchange experiences and establish a shared commitment later at home - Behavior strategy session for both parents and children to reinforce the acquisition of healthier physical activity and eating habits within the family - Extra activities to encourage and experience this more active behavior	- BMI score and BMI percentile - Food frequency questionnaire - ActiGraph GT3X + accelerometer	- The intervention group showed a the non-significant decrease in BMI score, however, increased PA, daily fruits servings, decreased daily soft drink consumption
Stark, 2014 [9]	Both the child and parents	*	*		Phase 1 (intensive intervention) - Modifying lifestyle behavior and improving parenting skills included educating on diet, PA, and goal setting of diet and PA - Group-based intervention for childhood included healthy eating, opportunities to try new foods and engage in PA - Home-based program included therapy modeling and parent rehearsal of dietary change	- BMI score and BMI percentile - 24-hour dietary recalls - Glucose tolerance test monitor GT1M accelerometer	- The intervention group showed a significant decreased on BMI score - The intervention showed a positive effect on decreasing calories intake, and increasing fruit and vegetable intake at home - The intervention also showed a level of moderate and vigorous PA
Taylor, 2018 [41]	Both the child and parents	*	*		Phase 1: Screening of health status and motivational interviewing for parents regarding child weight Phase 2 - Individualized feedback for parents about their child’s diet and PA - Single multidisciplinary consultant session and followed by brief contact about dietary goals) or approaches to parenting - Face-to-face session at home, and personalized support and problem solving	- BMI and z-scores - Accelerometer - Children dietary questionnaire - Lifestyle behaviors checklist	- The intervention group showed a significantly lower on BMI score, increasing fruits and vegetable intake as well as being more physically active after 24 months follow-up
van Nassau, 2014 [18]	Both the child and parents	*	*	*	- Implementing the Dutch Obesity Intervention in Teenagers (DOiT) Program that focused on increasing awareness and to inducing behavioral changes - Reducing intake of sugar-containing beverage - Reducing consumption of high-energy snack or sweet foods - Reducing screen time - Rising levels of activity such as active transport to school and sport participation - Consuming breakfast daily - Increasing social support of the parents and encouraging of the availability and accessibility of health product and activities in the home environment	- BMI and z-score - DOiT questionnaire	- The intervention group showed a significantly reduced on the BMI score and reduced sugar-containing beverage consumption
West, 2010 [8]	Both the child and parents	*	*	*	- Nutrition strategies such as establishing eating routines, modifying recipes, reading food labels - Physical activity activities such as reducing television and computer time, increasing energetic play, and encouraging involvement in sport - Positive parenting strategies such as keeping track of children’s lifestyle behaviors, setting clear guidelines about food and physical activity, and reinforcing healthy behaviors - Telephone follow-ups to review the implementation of strategies and problem solving - Final group session for technical and administrative support.	- BMI z-scores	- The intervention group had a significantly reduced in BMI z-score among children after 12 weeks follow-up
Xu, 2015 [26]	Both the child and parents	*	*	*	- Conducting a routine health education classes on healthy eating and physical activity - Encouraging children to adopt healthy eating including consuming less high-energy density foods such as fatty meat, fried snacks, and soft drinks - Advocating adolescents to increase time in moderate to vigorous physical activity and decreasing the sedentary behaviors such as watching TV and using the computer - Supporting school environments and family involvement	- BMI z-score - The Chinese version of the International Physical activity questionnaire - Food frequency questionnaire (FFQ)	- The intervention group showed a larger marginal reduction compared with the control group - The intervention group increased the frequency of jogging/running, decreased frequency of viewing TV as well as decreased sedentary time

Note: DB (dietary behaviors); PA (Physical Activity); SB (sedentary behaviors).

## References

[1] WHO (2018). Taking Action on Childhood Obesity Report.

[2] Reilly J.J., Kelly J. (2011). Long-term impact of overweight and obesity in childhood and adolescence on morbidity and premature mortality in adulthood: Systematic review. Int. J. Obes..

[3] Pigeot I., Barba G., Chadjigeorgiou C., de Henauw S., Kourides Y., Lissner L., Marild S., Pohlabeln H., Russo P., Tornaritis M., Veidebaum T., Wawro N., Siani A. (2009). Prevalence and determinants of childhood overweight and obesity in European countries: Pooled analysis of the existing surveys within the IDEFICS Consortium. Int. J. Obes..

[4] Tim L., Boyd S., McQueen D.V., Jones C.M. (2007). Health Promotion to Prevent Obesity In Global Perspectives on Health Promotion Effectiveness.

[5] Kreb N.F., Jacobson M.S., American Academy of Pediatrics Committee on Nutrition (2003). Prevention of Pediatric Overweight and Obesity. Pediatrics.

[6] Mulrin H. (2013). Interventions to Prevent Childhood Obesity Literature Review.

[7] Danford C.A., Schultz C., Marvicsin D. (2015). Parental roles in the development of obesity in children: Challenges and opportunities. Res. Rep. Biol..

[8] West F., Sanders M.R., Cleghorn G.J., Davies P.S. (2010). Randomised clinical trial of a family-based lifestyle intervention for childhood obesity involving parents as the exclusive agents of change. Behav. Res. Ther..

[9] Stark L.J., Clifford L.M., Towner E.K., Filigno S.S., Zion C., Bolling C., Rausch J. (2014). A pilot randomized controlled trial of a behavioral family-based intervention with and without home visits to decrease obesity in preschoolers. J. Pediatr. Psychol..

[10] Ostbye T., Krause K.M., Stroo M., Lovelady C.A., Evenson K.R., Peterson B.L., Bastian L.A., Swamy G.K., West D.G., Brouwer R.J., Zucker N.L. (2012). Parent-focused change to prevent obesity in preschoolers: Results from the KAN-DO study. Prev. Med..

[11] Croker H., Viner R.M., Nicholls D., Haroun D., Chadwick P., Edwards C., Wells J.C., Wardle J. (2012). Family-based behavioural treatment of childhood obesity in a UK National Health Service setting: Randomized controlled trial. Int. J. Obes..

[12] French S.A., Gerlach A.F., Mitchell N.R., Hannan P.J., Welsh E.M. (2011). Household obesity prevention: Take Action—A group-randomized trial. Obesity.

[13] Turner L.S.L., Altman D.G., Weeks L., Peters J., Kober T., Dias S., Schulz K.F., Plint A.C., Moher D. (2012). Consolidated standards of reporting trials (CONSORT) and the completeness of reporting of randomised controlled trials (RCTs) published in medical journals. Cochrane Database Syst. Rev..

[14] Taylor R.W., Cox A., Knight L., Brown D.A., Meredith-Jones K., Haszard J.J., Dawson A.M., Taylor B.J., Williams S.M. (2015). A Tailored Family-Based Obesity Intervention: A Randomized Trial. Pediatrics.

[15] Arauz Boudreau A.D., Kurowski D.S., Gonzalez W.I., Dimond M.A., Oreskovic N.M. (2013). Latino families, primary care, and childhood obesity: A randomized controlled trial. Am. J. Prev. Med..

[16] Gerards S.M., Dagnilie P.C., Jansen M.W., van der Goot L.O., de Vries N.K., Sanders M.R., Kremers S.P. (2012). Lifestyle Triple P: A parenting intervention for childhood obesity. BMC Public Health..

[17] Sacher P.M., Kolotourou M., Chadwick P.M., Cole T.J., Lawson M.S., Lucas A., Singhal A. (2010). Randomized controlled trial of the MEND program: A family-based community intervention for childhood obesity. Obesity.

[18] Van Nassau F.S.A., Cerin E., Salmon J., van Mechelen W., Brug J., Chinapaw M.J.M. (2014). The Dutch Obesity Intervention in Teenagers (DOiT) cluster controlled implementation trial: Intervention effects and mediators and moderators of adiposity and energy balance-related behaviours. Int. J. Behav. Nutr..

[19] Epstein L.H., Gordy C.C., Raynor H.A., Beddome M., Kilanowski C.K., Paluch R. (2001). Increasing fruit and vegetable intake and decreasing fat and sugar intake in families at risk for childhood obesity. Obes. Res..

[20] Burrows T.W.J., Collins C.E. (2010). The impact of a child obesity treatment intervention on parent child-feeding practices. Int. J. Pediatr. Obes..

[21] Knowlden A.P., Sharma M. (2012). Systematic review of family and home-based interventions targeting paediatric overweight and obesity. Obes. Rev..

[22] Bruno A., Escobar P., Cebolla A., Alvarez-Pitti J., Guixeres J., Lurbe E., Banos R., Lison J.F. (2018). Home-exercise Childhood Obesity Intervention: A Randomized Clinical Trial Comparing Print Versus Web-based (Move It) Platforms. J. Pediatr. Nurs..

[23] Cao Z.J., Wang S.M., Chen Y. (2015). A randomized trial of multiple interventions for childhood obesity in China. Am. J. Prev. Med..

[24] Elder J.P., Crespo N.C., Corder K., Ayala G.X., Slymen D.J., Lopez N.V., Moody J.S., McKenzie T.L. (2014). Childhood obesity prevention and control in city recreation centres and family homes: The MOVE/me Muevo Project. Pediatric Obes..

[25] Gallotta M.C., Iazzoni S., Emerenziani G.P., Meucci M., Migliaccio S., Guidetti L., Baldari C. (2016). Effects of combined physical education and nutritional programs on schoolchildren’s healthy habits. PeerJ.

[26] Xu F., Ware R.S., Leslie E., Tse L.A., Wang Z., Li J., Wang Y. (2015). Effectiveness of a Randomized Controlled Lifestyle Intervention to Prevent Obesity among Chinese Primary School Students: CLICK-Obesity Study. PLoS ONE.

[27] Colley R.C., Brownrigg M., Tremblay M.S. (2012). A model of knowledge translation in health: The Active Healthy Kids Canada Report Card on physical activity for children and youth. Health Promot. Pract..

[28] Gruber K.J., Haldeman L. (2009). Using the family to combat childhood and adult obesity. Prev. Chronic. Dis..

[29] Spear B.A., Barlow S.E., Ervin C., Ludwig D.S., Saelens B.E., Schetzina K.E., Taveras E.M. (2007). Recommendations for treatment of child and adolescent overweight and obesity. Pediatrics.

[30] Lison J.F., Real-Montes J.M., Torro I., Arguisuelas M.D., Alvarez-Pitti J., Martinez-Gramage J., Aguilar F., Lurbe E. (2012). Exercise intervention in childhood obesity: A randomized controlled trial comparing hospital-versus home-based groups. Acad. Pediatrics.

[31] Larsen K.T., Huang T., Ried-Larsen M., Andersen L.B., Heidemann M., Moller N.C. (2016). A Multi-Component Day-Camp Weight-Loss Program Is Effective in Reducing BMI in Children after One Year: A Randomized Controlled Trial. PLoS ONE.

[32] Farias Edos S., Goncalves E.M., Morcillo A.M., Guerra-Junior G., Amancio O.M. (2015). Effects of programmed physical activity on body composition in post-pubertal schoolchildren. J. Pediatr..

[33] Baranowski T.T.D., Buday R., Lu A.S., Baranowski J. (2010). Design of Video Games for Children’s Diet and Physical Activity Behavior Change. Int. J. Comput. Sci. Sport..

[34] Atkin A.J., Gorely T., Clemes S.A., Yates T., Edwardson C., Brage S., Salmon J., Marshall S.J., Biddle S.J. (2012). Methods of Measurement in epidemiology: Sedentary Behaviour. Int. J. Epidemiol..

[35] American Academic of Paediatrics (2001). American Academy of Pediatrics: Children, adolescents, and television. Paediatrics.

[36] Harder-Lauridsen N.M., Birk N.M., Ried-Larsen M., Juul A., Andersen L.B., Pedersen B.K., Krogh-Madsen R. (2014). A randomized controlled trial on a multicomponent intervention for overweight school-aged children—Copenhagen, Denmark. BMC Pediatr..

[37] Markert J., Herget S., Petroff D., Gausche R., Grimm A., Kiess W., Bluher S. (2014). Telephone-based adiposity prevention for families with overweight children (T.A.F.F.-Study): One year outcome of a randomized, controlled trial. Int. J. Environ. Res. Public Health.

[38] Patrick K., Norman G.J., Davila E.P., Rosenberg D.E., Calfas K.J., Covin J., Sallis J.F. (2013). Two-Year Outcomes of a Primary Care–and Home-Based Intervention for Physical Activity, Sedentary Behavior, and Diet in Adolescents. ICAN Infant Child Adolesc. Nutr..

[39] Serra-Paya N., Ensenyat A., Castro-Vinuales I., Real J., Sinfreu-Bergues X., Zapata A., Mur J.M., Galindo-Ortego G., Sole-Mir E., Teixido C. (2015). Effectiveness of a Multi-Component Intervention for Overweight and Obese Children (Nereu Program): A Randomized Controlled Trial. PLoS ONE.

[40] Crespo N.C., Elder J.P., Ayala G.X., Slymen D.J., Campbell N.R., Sallis J.F., McKenzie T.L., Baquero B., Arredondo E.M. (2012). Results of a multi-level intervention to prevent and control childhood obesity among Latino children: The Aventuras Para Ninos Study. Ann. Behav. Med. A Publ. Soc. Behav. Med..

[41] Taylor S.F.Y., Solomon S. (2017). Factors affecting the self-monitoring of blood glucose levels in Aboriginal patients: Findings from a remote community. Aust. Indig. Health.

[42] Ash T., Agaronov A., Young T., Aftosmes-Tobio A., Davison K.K. (2017). Family-based childhood obesity prevention interventions: A systematic review and quantitative content analysis. Int. J. Behav. Nutr. Phys. Act..

[43] Berge J.M., Everts J.C. (2011). Family-Based Interventions Targeting Childhood Obesity: A Meta-Analysis. Child. Obes..

[44] Bleich S.N., Vercammen K.A., Zatz L.Y., Frelier J.M., Ebbeling C.B., Peeters A. (2018). Interventions to prevent global childhood overweight and obesity: A systematic review. Lancet Diabetes Endocrinol..

[45] Showell N.N., Fawole O., Segal J., Wilson R.F., Cheskin L.J., Bleich S.N., Wu L., Lau B., Wang Y. (2013). A systematic review of home-based childhood obesity prevention studies. Pediatrics.

